# Rhinovirus infection and co-infection in children with severe acute respiratory infection during the COVID-19 pandemic period

**DOI:** 10.1080/21505594.2024.2310873

**Published:** 2024-02-21

**Authors:** Célia Regina Malveste Ito, Mônica Oliveira Santos, Marcos de Oliveira Cunha, Kelliane Martins de Araújo, Guilherme Rocha Lino de Souza, Geovana Sôffa Rézio, Pollyanna Neta de Brito, Alana Parreira Costa Rezende, Jakeline Godinho Fonseca, Isabela Jubé Wastowski, José Daniel Gonçalves Vieira, Melissa Ameloti Gomes Avelino, Lilian Carla Carneiro

**Affiliations:** a Microorganism Biotechnology Laboratory of Institute of Tropical Pathology and Public Health, Federal University of Goiás– 235 St. Leste Universitário neighborhood, Goiânia, Goiás, Brazil; b Biochemistry and Molecular Biology Laboratory, Biologic Science Institute, Federal University of Goiás, Samambaia Camp, Goiânia, Goiás, Brazil; c State Emergency Hospital of the Northwest Region of Goiânia Governador Otávio Lage de Siqueira (HUGOL), Goiânia, Goiás, Brazil; d Molecular Immunology Laboratory of Goiás State University, Laranjeiras Unity Prof. Alfredo de Castro neighborhood, Goiânia, Goiás, Brazil; e Department of Pediatrics, Federal University of Goiás, Universitaria Avenue, Leste Universitário neighborhood, Goiânia, Goiás, Brazil

**Keywords:** Virus, epidemiology, microbiology, pandemic, child, several infections

## Abstract

Rhinovirus causes respiratory tract infections in children and is found in co-infections. The objective of this research was to study the clinical profile of rhinovirus infection and co-infection in children with severe acute respiratory infection (SARI) during the COVID-19 pandemic period. We included 606 children ranging in age from 0.1 to 144 months of age from March 2020 to December 2021, hospitalized in the Pediatric Intensive Care Unit (PICU). The samples were collected by secretion from the nasopharynx region. A total of 259 children were tested positive for viral infection, 153 (59.07%) of them had a single rhinovirus infection and, 56 (36.6%) were aged between 60.1 and 144 months. Nine types of co-infections were identified and were found coinfection with three or more viruses (22/104, 21.15%). Observing the seasonality, the number of cases was similar between 2020 (49.53%) and 2021 (51.47%). Patients with a single infection (86.88%) and coinfection (67.30%) were more likely to have coughed. Patients with co-infection required the use of O2 for longer than those with a single rhinovirus infection. Hemogram results obtained from individuals with a single infection had higher levels of urea when compared to patients with co-infection with and other respiratory viruses. Multiple correspondence analyses indicated different clinical symptoms and comorbidities in patients with co-infection compared to those with single infection. The results found that the rhinovirus was much prevalent virus during the pandemic period and was found in co-infection with other virus types, what is important to diagnostic for the correct treatment of patients.

## Introduction

Since antiquity the world has experienced several deaths due to various viruses, claiming many lives from adults to children. At the end of 2019, the world began fighting a new virus that generated a pandemic, SARS-CoV-2, which causes Coronavirus Disease 2019 (COVID-19), and currently represents one of the largest public health emergencies in history [1]. Approximately 68.4 million people worldwide acquired COVID-19, with about 45 million people recovering from the virus (±65.6%) and more than 1.5 million died (±2.28) from the disease [1,2].

Respiratory viruses such as influenza, para-influenza, respiratory syncytial virus (RSV), human metapneumovirus (hMPV), human rhinovirus (hRV) and human coronavirus are reported as the main responsible for respiratory tract infections in children, before the COVID-19 pandemic [3–5]. Data from some studies have shown a reduction in the prevalence of other seasonal respiratory viruses due to preventive measures for COVID-19 [6–8]. It is not yet well defined whether national mitigation strategies, including isolation and social distancing, suspension of classes, and mandatory use of face mask would change the course of circulation of viral pathogens during COVID-19, as well as co-infections of acute severe respiratory syndrome coronavirus-2 (SARS-CoV-2) and other respiratory pathogens [9–13].

Rhinovirus is the most common agent that causes respiratory tract infections in children and is found in mixed viral–bacterial and viral–viral infections, as well as in large proportion in PCR samples from upper respiratory tract samples in asymptomatic children [14–16]. Belonging to the genus Enterovirus, the hRVs are very small viruses containing a non-enveloped RNA classified into three species (A, B and C) and based on the antigenic and genetic characteristics, they have approximately 160 different types, which circulate simultaneously in populations throughout the year explaining the high frequency of infections by this pathogen [14,17,18].

The aim of this research was to study the clinical profile of rhinovirus infection and co-infection in children with severe acute respiratory infection (SARI) during the COVID-19 pandemic period.

## Methods

We included 606 children ranging in age from 0.1 to 144 months of age, with severe acute respiratory infections (SARI) from March 2020 to December 2021, hospitalized in the Pediatric Intensive Care Unit (PICU) for respiratory diseases of the Otávio Lage de Siqueira Emergency Hospital (HUGOL) being the state reference for cases of COVID-19 by the Public Health System (SUS). All patients included were followed up until the outcome of the clinical picture of SARI hospitalized in the PICU. Table 1 shows the rate of SARI in children during COVID-19 pandemic in Goiás-Brazil., in the years 2020 and 2021 [19].

**Table 1.** SARI cases in children in the state of Goiás-Brazil, in the years 2020 and 2021.

YEARS	2020				2021			
SARI/AGE	<2	2 to 4	5 to 9	10 to 19	<2	2 to 4	5 to 9	10 to 19
In investigation	0	0	3	0	11	10	1	4
Not specified	541	251	236	335	656	336	274	314
By COVID-19	112	43	52	107	183	98	69	389
By Influenza	5	3	1	3	13	11	16	12
By other respiratory viruses	144	80	45	16	439	167	95	10
By other pathogens	1	1	5	4	4	0	1	2

For viral identification, 606 samples were collected with Rayon swabs of secretion from the nasopharynx region of the 606 hospitalized children. The swabs were stored in 15 mL Falcon tubes containing 3 mL of viral transport medium (MTV) and sent to the laboratory. The samples were transferred to tubes to transport samples of 5 mL containing 750 μL of TRIzol-LS reagent (Invitrogen) and 250 μL of the sample collected, then the tubes with the samples were stored in freezer −80°C until the moment of ribonucleic extraction.

The amplification of the genetic material was performed with the kits of the Thermo Fisher Scientific® (Stockholm, Sweden) (Applied Biosystems TrueMarkTM Respiratory I Catalog number [A56284C] and Applied Biosystems TrueMarkTM Respiratory II Catalog number [A56286C]). Both kits contained primer/probe sets specific to genomic regions of rhinovirus, coronavirus (SARS-CoV-2); Syncytial virus bocavirus, adenovirus influenza, Metapneumovirus, parainfluenza and SARS-Like. For the positive control of the reaction was used the RNAseP gene amplification, this protein is specifically found in human organisms. For the amplification of the RNAseP genetic material was used the kits of the Thermo Fisher Scientific ® (Stockholm, Sweden) (Applied Biosystems TrueMarkTM Respiratory I Catalog number [4316831]). For the negative control of the reaction was added water in place of the genetic material. The protocol of reaction and the qPCR conditions were done according to the fabricant instructions.

A standard form with information about demographics and clinical presentation was submitted. The project was approved by the Ethics Committee of the Hospital das Clinicas of the Federal University of Goiás, it seems CAAE: 33540320.7.0000.5078.

### Statistical analysis

Descriptive and comparative analyses were performed in categorical data by viral infection status (single infection or co-infection). The χ2 test was used to compare categorical variables between different groups. Multiple correspondence analyses were performed to verify the relationship between single rhinovirus infection and co-infection with other respiratory viruses, age of patients and the different types of co-infections with the clinical characteristics and comorbidities of patients. Normality and homoscedasticity tests were performed to verify the distribution of quantitative data. Subsequently, nonparametric tests (Kruskal Wallis and Man Whitney a posteriori) were performed to verify the relationship between quantitative variables. A *p* value <0.05 was considered statistically significant. The data were analyzed using the Software R Studio, version 4.2.2.

## Results

### Patient characteristics

A total of 259 children were tested positive for viral infection, 153 (59.07%) of them had a single viral infection (Rhinovirus), while 106 (40.93%) were diagnosed with viral co-infection. Among the children with infection only with hRV, 81 (52.9%) were boys and 72 (47.1%) were girls; 51 (33.3%) aged between 0.1 and 24 months, 46 (30.06%) were aged between 24.1 and 60 months, and 56 (36.6%) were aged between 60.1 and 144 months.

Similar to single infection with hRV (p > 0,05), among children presenting co-infection between hRV and another respiratory virus, 61 (57.5%) were boys, while 45 (42.5%) were girls, 40 (38.6%) aged between 0 and 24 months, 36 aged between 24.1 and 60 months (33.9%), while the remaining 28 (26.4%) were aged between 60,1 and 144 months (Table 2).

**Table 2.** Baseline characteristics of children with a single viral infection and co-infection.

Characteristics	Single infection (Rhinovírus; *n* = 153)	Coinfection (*n* = 106)	*p* valour
Age months (%)			0.227
0.1 to 24	33.3%	38.6%	
24.1 to 60	30.06%	33.9%	
60.1 a 144	36.6%	26.4%	
Sex (%)			0.545
Male	52.9%	57.5%	
Female	47.1%	42.5%	
Comorbidities			
Chronic asthma	30.71%	16.03%	0.011
Genetic disease	5.26%	4.71%	0.927
Heart disease	4.71%	1.3%	0.203
Neurological disease	14.15%	5.22%	0.024
Others	21.69%	7.84%	0.003

As for the presence of comorbidities, a similar number of patients with genetic and cardiac diseases were associated with a single infection and co-infection. Patients with chronic asthma were more prevalent among patients with a single infection (*p* < 0.05; Table 2); however, a greater number of patients with co-infections had neurological diseases and other comorbidities (i.e. prematurity, malnutrition, anemia sickle cell, burns).

### Proportion and seasonality of viruses detected

**Figure 1.**
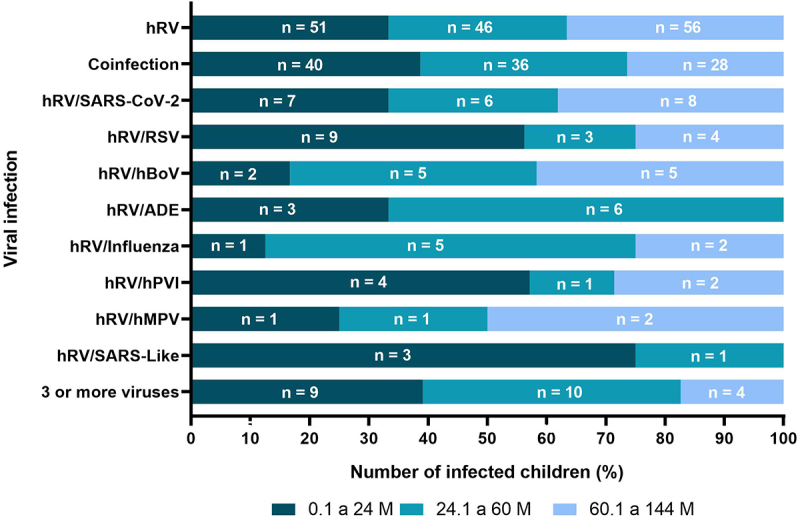
Age range of children with a viral condition with a single infection and children with co-infection with and another virus. Abbreviations: M (months), (human rhinovirus), hBoV (bocavirus), SARS-CoV-2 (coronavirus), hMPV (metapneumovirus), hPVI (parainfluenza), RSV (respiratory syncytial virus).

Nine types of co-infections were identified: (i)/Coronavirus (SARS-CoV-2); (ii)/Respiratory Syncytial Virus (RSV); (iii)/Bocavirus (hBoV); (iv)/Adenovirus (ADE); (v)/Influenza; (vi)/Metapneumovirus (hMPV); (vii)/Parainfluenza (hPVI); (viii)/SARS-Like; (ix) co-infection with three or more viral types (Figure 1). Among the coinfections were found coinfection with three or more viruses (22/104, 21.15%)/SARS-CoV-2 (21/104; 20.19%)/RSV (15/104, 14.4%), and/hBoV (12/104, 11.53%). The most prevalent were: /ADE (9/104; 8.65%)/Influenza (8/104; 7 .69%)/hPVI (7/104; 6.73%)/hMPV (4/104; 3.84%), and/SARS-Like (4/104; 3.84%) (Figure 1).

**Figure 2.**
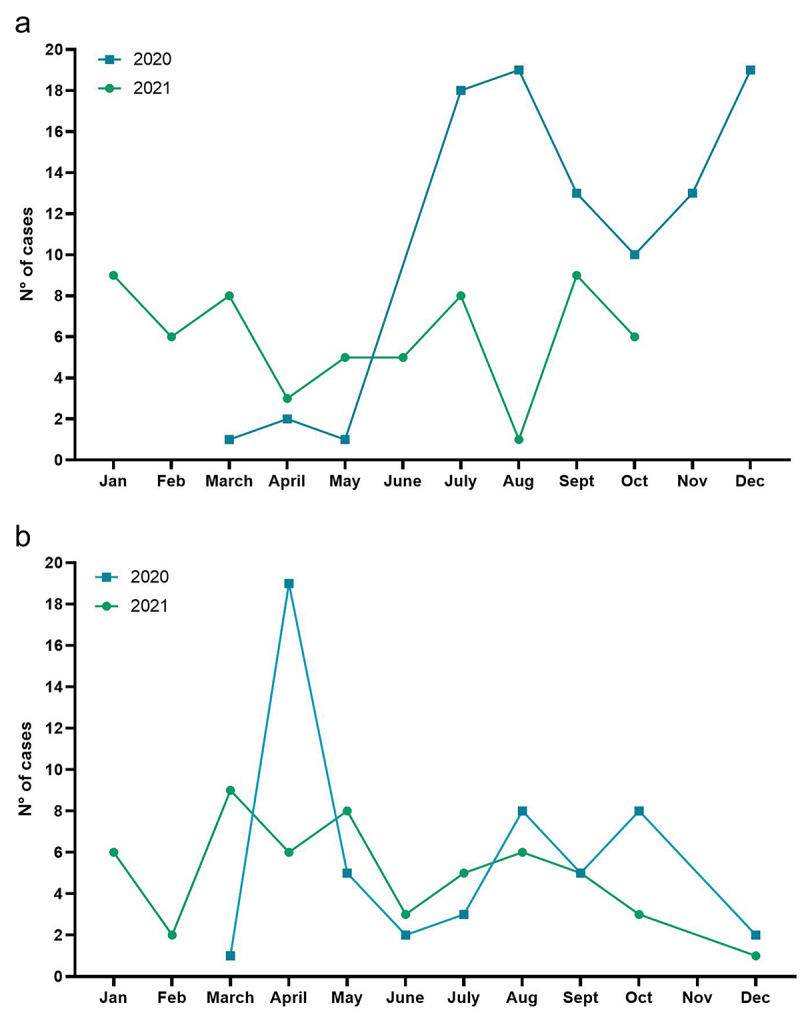
Seasonality of Rhinovirus (a) and co-infections with Rhinovirus (b) in 2020 and 2021.

Most cases of rhinovirus infection occurred in 2021 (61.53%) with prevalence between July and December 2021, with peaks of infection in July (18.75%), August (19.79%) and December (19.79%), while 38.47% of cases occurred between January and October 2021, with peaks of infection in January (15%), March (13.33%), July (13.33%) and September (15%) (Figure 2(a)). When observing the seasonality of co-infection cases, the number of cases was similar between 2020 (49.53%) and 2021 (51.47%), with infection peaks in April (35.84%), August (15.09%) and October (15.09%) of 2020, and in March (16.66%), May (14.81%) and August (15.09%) of 2021 (Figure 2(b)).

### Clinical results

Patients with a single infection were more likely to have cough, dyspnea/tachypnea, pneumonia, bronchiolitis and need for O2 use (*p* < 0.05), when compared to patients with co-infection with and another virus respiratory. However, patients with viral co-infection were more likely to have fever, runny nose/rhinorrhea, abnormal chest X-rays, and need for intermittent mandatory ventilation (*p* < 0.05; Table 3, Figure 1 supplementary data).

**Table 3.** Clinical outcomes of children with single viral infection with hRV, and co-infection with and other respiratory viruses.

Characteristics	Rhinovirus single infection (*n* = 153)	Co-infection (*n* = 106)	χ^2^	*p* valour
Fever	40.16%	54.80%	4.264	0.039
RN/R	41.80%	63.46%	9.698	0.002
Cough	86.88%	67.30%	11.379	< 0.001
Sneezing	9.61%	14.75%	0.933	0.34
Wheezing	40.38%	51.63%	2.709	0.1
D/T	86.88%	50.96%	33.036	< 0.001
PNM	29.41%	13.97%	6.814	0.009
AVB	57.51%	15.09%	45.145	< 0.001
O_2_ Use	79.50%	64.07%	5.911	0.015
altered chest X-ray	36.60%	56.73%	9.349	0.003
NIV	15.57%	18.44%	0.156	0.693
IMV	6.55%	16.5%	4.634	0.031
Death	3.92%	6.12%	0.244	0.621

Abbreviations: M (months), RN/R (runny nose/rhinorrhea), D/T (dyspnea/tachypnoea), PNM (pneumonia), AVB (acute viral bronchiolitis), NIV (Non-Invasive Ventilation), IMV (Intermittent Mandatory Ventilation).

**Figure 3.**
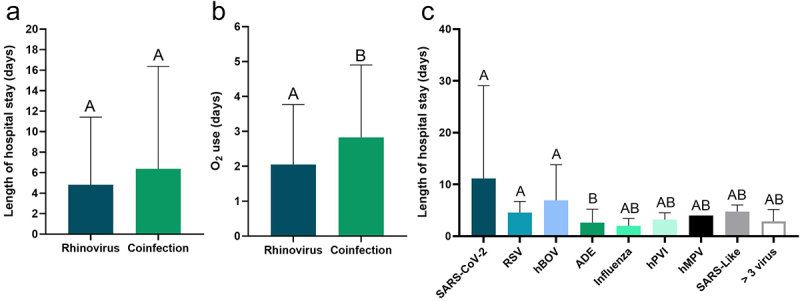
Length of stay (a, c) and length of O2 use (b) of patients with a single infection with hRV and with different co-infections and other respiratory viruses. SARS-VoV-2 (coronavirus 2); RSV (respiratory syncytial virus); ADE (Adenovirus); hPVI (Parainfluenza); hMPV (metapneumovirus); SARS-Like (coronaviruses).

An analysis was carried out considering the time of interaction according to the viral conditions and, even though the length of hospital stay was similar among the viral conditions (*p* > 0.05); (Figure 3(a)), patients with co-infection required the use of O2 for longer than those with a single rhinovirus infection (*p* < 0.05); (Figure 3(b)). Figure 3(c) shows the length of hospital stay for co-infection with hRV and other viruses, with hRV/SRAS-CoV-2 co-infection having the longest length of stay, followed by hRV/hBoV.

**Figure 4.**
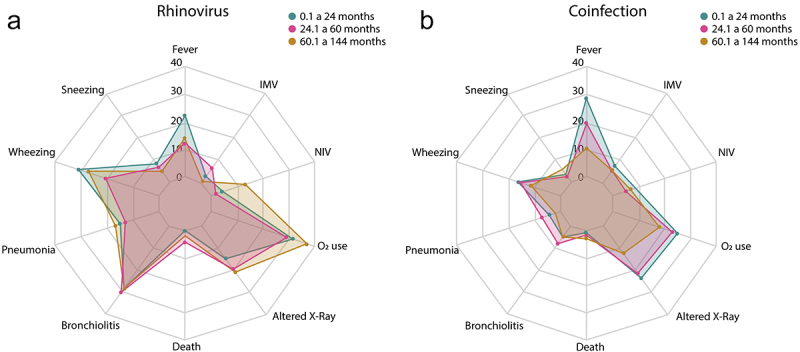
Clinical outcomes of children with single viral infection with hRV (a), and co-infection with another respiratory virus (b) according to their age.

The propensities for clinical symptoms were age-dependent in patients with a single infection only for coryza/rhinorrhea, with a higher incidence in patients aged 0.1 to 24 months (*p* < 0.05), the use of non-invasive ventilation, with a higher incidence in patients aged 60.1 to 144 months (*p* < 0.05), and intermittent mandatory ventilation, with a higher incidence in patients aged 0.1 to 24 and 24.1 to 60 months (*p* < 0.05), while for patients with co-infection only fever was more likely to occur in patients aged 0.1 to 24 months (*p* < 0.05; Table 4, Figure 4).

**Table 4.** Clinical outcomes of patients with single viral infection with hRV and co-infection according to their age.

	Single infection (*n* = 153)			Co-infection (*n* = 106)				
Charac.	0.1–24 M	24.1–60 M	60–144 M	0.1–24 M	24.1–60 M	60–144 M	χ^2^	*p* valor
Fever	51.16%	32.5%	35.89%	68.29%	54.28%	35.71%	16.15	0.006
RN/R	72.09%	55%	46.15%	29.26%	48.57%	32.14%	19.41	0.002
Cough	88.37%	80%	92.30%	75.61%	68.57%	53.57%	19.05	0.002
Sneezing	18.60%	15%	10.25%	7.31%	5.71%	17.85%	5.12	0.401
Wheezing	60.78%	45.65%	48.21%	39.02%	42.85%	39.28%	5.91	0.315
D/T	79.07%	82.5%	100%	51.22%	48.57%	53.57%	39.77	< 0.001
PNM	29.41%	28.26%	30.35%	9.75%	18.91%	7.14%	12.17	0.033
AVB	58.82%	65.21%	50%	12.19%	21.62%	10.71%	50.40	< 0.001
O_2_ Use	60.976	67.647	64.286	72.093	94.872	72.500	13.85	0.017
altered chest X-ray	29.41%	43.47%	37.5%	58.53%	62.85%	46.42%	13.89	0.016
NIV	9.30%	5%	33.33%	17.07%	14.70%	25%	14.73	0.012
IMV	4.65%	15%	0%	17.07%	14.70%	17.85%	10.51	0.062
Death	0%	8.6%	3.57%	2.63%	6.25%	10.71%	6.992	0.221

Abbreviations: Charac. (Characteristic), M (months), RN/R (runny nose/rhinorrhea), D/T (dyspnea/tachypnea), PNM (pneumonia), AVB (acute viral bronchiolitis), NIV (Non-Invasive Ventilation), IMV (Intermittent Mandatory Ventilation).

When comparing patients with single infection and co-infection according to age, the patients aged 0.1 to 24 months with a single infection, were more prone to runny nose/rhinorrhea, cough, dyspnea/tachypnea, pneumonia, and bronchiolitis (*p* < 0.05), from 24.1 to 60 months were more prone to dyspnea/tachypnea and bronchiolitis (*p* < 0.05), and from 60.1 to 144 months were more prone to cough, dyspnea/tachypnea, pneumonia and bronchiolitis (*p* < 0. 05) when compared to patients with viral co-infection with the same age group (0.1 to 24, 24.1 to 60 and 60.1 to 144 months) (Figure 4).

However, patients aged 0.1 to 24 months with co-infection, were more likely to have changes in chest X-rays (*p* < 0.05); patients aged 60.1 to 144 months had a greater need for O2 and intermittent mandatory ventilation when compared to patients aged 0.1 to 24 and 60.1 to 144 months with a single infection, respectively (*p* < 0.05) (Figure 4).

When observing the different types of co-infections separately, it is possible to observe similar clinical symptoms in general (Table 5, Figure 2 Supplementary Data), but with some differences related to the greater induction of these symptoms among some infections: (i) the length of hospital stay was greater in patients with hRV/SARS-CoV2, hRV/RSV and hRV/hBoV co-infections compared to hRV/ADE co-infection (*p* < 0.05); (ii) fever was more prevalent in patients co-infected with more than three viruses compared to hRV/ADE co-infection (*p* < 0.05); (iii) runny nose/rhinorrhea was more prevalent in patients affected by hRV/hBoV compared to hRV/SARS-Like, hRV/RSV and co-infections with more than three viruses; (iv) cough was more prevalent in patients co-infected with more than 3 viruses compared to hRV/SARS-Like co-infection (*p* < 0.05); (v) sneezing was more prevalent in patients co-infected with more than three viruses compared to hRV/PVI co-infection; (vi) wheezing was more prevalent in patients with hRV/hBoV and hRV/SARS-Like co-infections compared to hRV/SARS-CoV2 and with more than 3 viruses, and hRV/SARS-CoV2 respectively; (vii) altered x-rays were more prevalent in patients affected by hRV/SARS-Like co-infection compared to hRV/PVI co-infection.

**Table 5.** Clinical results of patients according to viral co-infection with rhinovirus.

Characteristic	SARS-CoV-2	RSV	hBoV	ADE	Influenza	hPVI	hMPV	SARS-Like	>3 viruses	χ^2^	P valour
Fever	20%	50%	66.66%	25%	25%	28.57%	75%	20%	73.91%	21.92	0.009
RN/R	26.08%	18.75%	66.66%	62.5%	62.5%	40%	25%	0%	26.08%	25.01	0.003
Cough	55%	68.75%	58.33%	87.5%	62.5%	71.42%	75%	20%	86.95%	28.09	< 0.001
Sneezing	10%	6.25%	25%	0%	12.5%	42.85%	0%	0%	0%	14.63	0.101
Wheezing	20%	43.75%	75%	50%	37.5%	28.57%	25%	80%	30.43%	17.79	0.038
D/T	40%	43.75%	66.66%	62.5%	12.5%	28.57%	75%	80%	60.87%	50.38	< 0.001
PNM	75%	68.75%	66.66%	62.5%	57.14%	28.57%	50%	100%	56.52%	17.17	0.046
AVB	9.52%	12.5%	16.66%	22.22%	12.5%	14.28%	25%	0%	8.69%	12.08	0.208
O_2_ Use	14.28%	31.25%	16.66%	22.22%	0%	0%	0%	40%	8.69%	51.68	< 0.001
Altered chest X-ray	70%	50%	58.33%	37,5%	37.5%	14.28%	75%	100%	60.87%	23.39	0.005
NIV	30%	12.5%	50%	0%	14.28%	0%	0%	0%	17.39%	17.04	0.048
IMV	35%	18.75%	25%	0%	0%	0%	25%	0%	13.04%	21.56	0.01
Death	5%	6.66%	16.66%	0%	0%	0%	25%	0%	4.76%	8.905	0.446

Abbreviations: M (months), RN/R (runny nose/rhinorrhea), D/T (dyspnea/tachypnoea), PNM (pneumonia), AVB (acute viral bronchiolitis), NIV (Non-Invasive Ventilation), IMV (Intermittent Mandatory Ventilation). RSV (Respiratory Syncytial virus); hBoV (Bocavirus); ADE (Adenovirus); hPVI (parainfluenza); hMPV (metapneumovirus)

### Blood count

**Figure 5.**
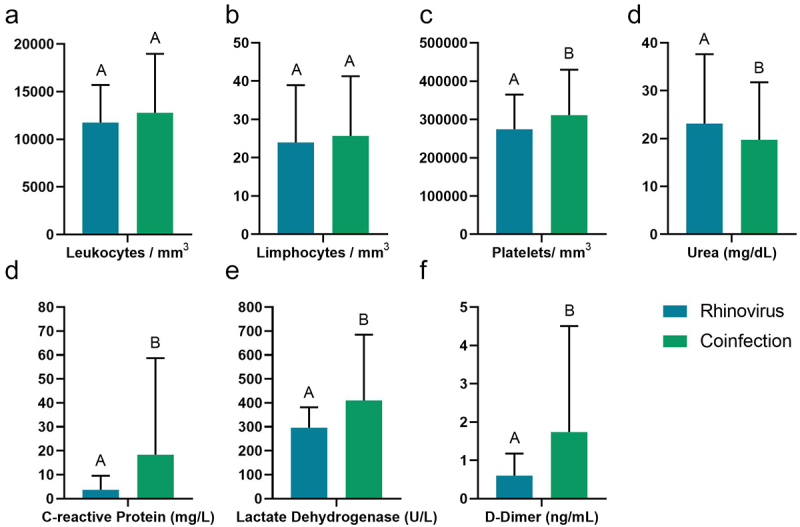
Blood count of patients with single infection and co-infections with other respiratory viruses. Abbreviations: CRP (C-reactive protein), LDH (lactate dehydrogenase).

Hemogram results obtained through the blood count of the patients, individuals with a single infection had higher levels of urea when compared to patients with co-infection by other respiratory viruses (*p* < 0.05; Figure 5); however, patients with viral co-infection showed elevated levels of platelets, C-reactive protein (CRP), lactate dehydrogenase (LHL) and D-dimer compared to those with single infection (*p* < 0.05; Figure 5).

**Figure 6.**
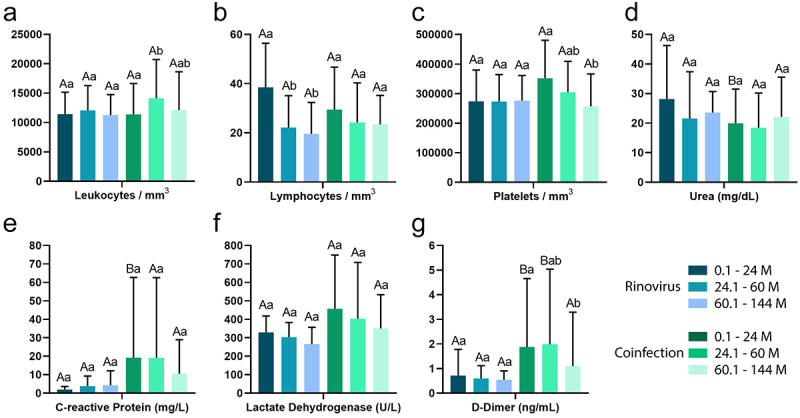
Blood count of patients with single infection and co-infections with other respiratory viruses according to their age. Abbreviations: M (months), CRP (C-reactive protein), LDH (lactate dehydrogenase).

Blood count results were age dependent only for lymphocytes in patients with a single infection, where children aged 0.1 to 24 months had higher levels of lymphocytes compared to children aged 24.1 to 60 and 60.1 to 144 months (*p* < 0. 05; Figure 6), while patients with viral co-infection had: (i) higher levels of leukocytes in patients aged 24.1 to 60 months; (ii) lower platelet levels in patients aged 60.1 to 144 months; (iii) and lower levels of D-Dimer in patients with 60.1 to 144 M (*p* < 0.05; Figure 6).

In addition, when comparing patients of the same age with a single infection and viral co-infection, patients aged 0.1 to 24 months with co-infection had higher levels of CRP, and those aged 0.1 to 24 and 24.1 to 60 months had higher levels of D-Dimer than those with a single infection (*p* < 0.05; Figure 6), while only patients aged 0.1 to 24 months with a single infection had higher urea levels than those with co-infection *(p* < 0.05; Figure 6).

**Figure 7.**
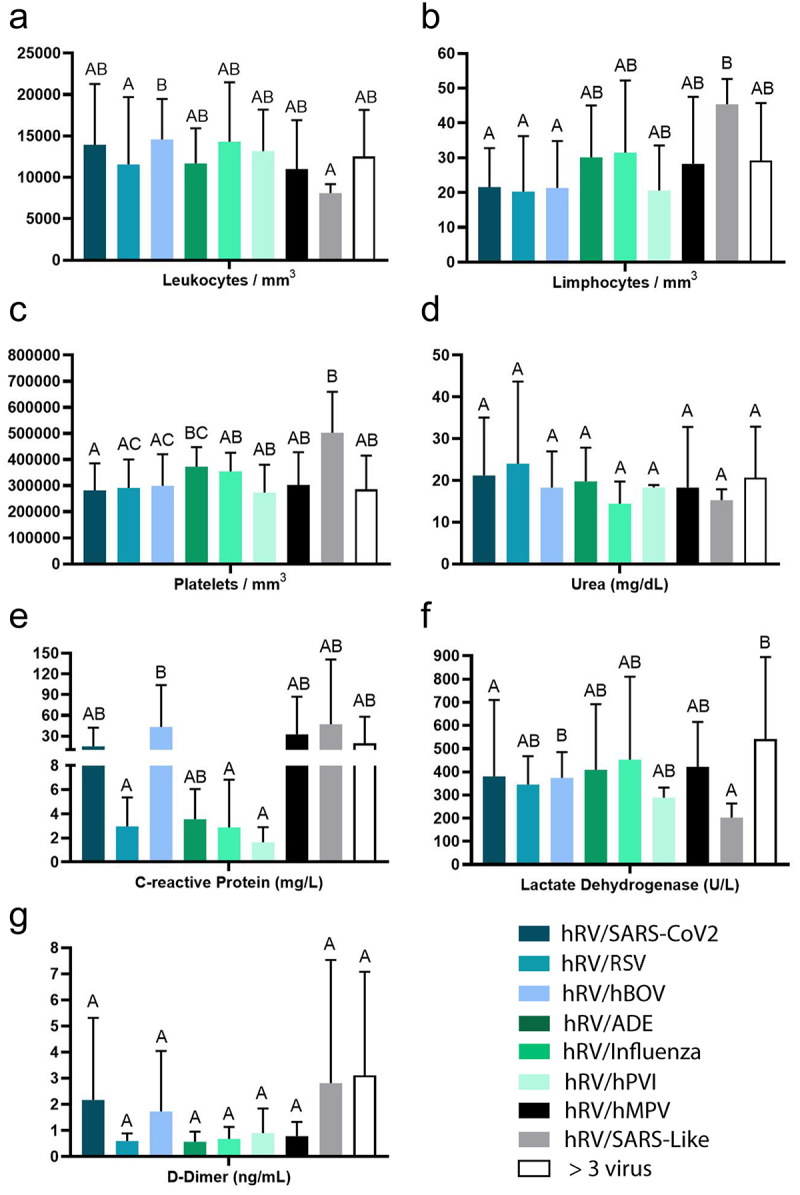
Blood count of patients with co-infections with and another respiratory virus. Abbreviations: CRP (C-reactive protein), LDH (lactate dehydrogenase). hRV/BoV (Rhinovirus/Bocavirus); hRV/SARS-CoV-2 (Rhinovirus/Coronavirus 2); hRV/SARS-Like (Rhinovirus/Coronaviruses); hRV/RSV (Rhinovirus/Syncytial); hRV/Influenza (Rhinovirus/Influenza); hRV/ADE (Rhinovirus/Adenovirus); hRV/hPVI (Rhinovirus/parainfluenza); hRV/MPV (Rhinovirus/Metapneumovirus).

When observing the blood count of patients with different co-infections (Figure 7), some differences were observed: (i) patients with hRV/SARS-Like had lower levels of leukocytes than those with hRV/hBoV; (ii) patients with hRV/SARS-Like had higher levels of lymphocytes than those with hRV/SARS-CoV2, hRV/RSV, hRV/hBoV; (iii) patients with hRV/SARS-Like had higher platelet levels than those with hRV/SARS-CoV2, hRV/RSV and hRV/hBoV; (iv) patients with hRV/hBoV had higher CRP levels than those with hRV/RSV/Influenza and hRV/hPVI; (v) hRV/hBoV had higher levels of LDH than those with hRV/SARS-Like, and lower levels of LDH than those with hRV/SARS-CoV2.

### Multiple correspondence analyses (MCA)

**Figure 8.**
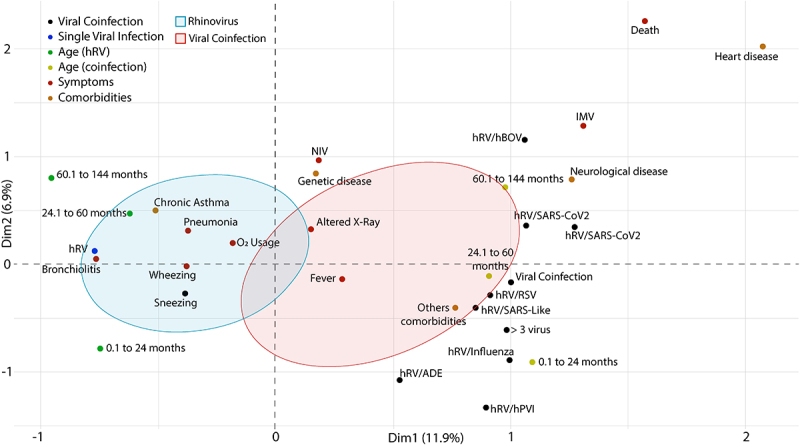
Multiple correspondence analyses (MCA) of patients with a single rhinovirus infection and co-infections with another respiratory virus. Abbreviations: C/R (runny nose/rhinorrhea), D/T (dyspnea/tachypnea), NIV (non-invasive ventilation), VMI (intermittent mandatory ventilation). hRV/BoV (rhinovirus/Bocavirus); hRV/SARS-CoV-2 (rhinovirus/Coronavirus 2); hRV/SARS-Like (rhinovirus/Coronaviruses); hRV/RSV (rhinovirus/Syncytial); hRV/Influenza (rhinovirus/Influenza); hRV/ADE (rhinovirus/Adenovirus); hRV/hPVI (rhinovirus/parainfluenza); hRV/MPV (rhinovirus/Metapneumovirus).

Multiple correspondence analyses (MCA) indicated different clinical symptoms and comorbidities in patients with co-infection compared to those with single infection (Figure 8).

**Figure 9.**
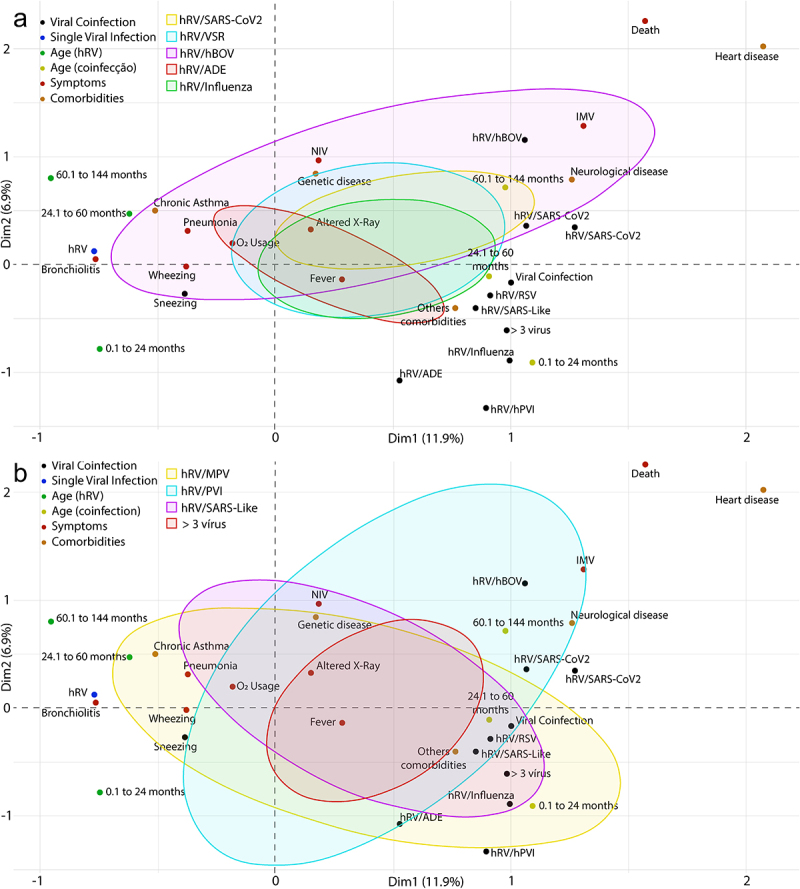
Multiple correspondence analysis (MCA) of patients with another respiratory virus. a - MCA of hRV/SARS-CoV2, hRV/RSV, hRV/hBoV, hRV/Influenza coinfections. b - MCA of co-infections hRV/MPV, hRV/PVI, hRV/SARS-Like, co-infection with more than three viruses (> three viruses). Abbreviations: C/R (runny nose/rhinorrhea), D/T (dyspnea/tachypnea), NIV (non-invasive ventilation), VMI (intermittent mandatory ventilation). hRV/BoV (Rhinovirus/Bocavirus); hRV/SARS-CoV-2 (Rhinovirus/Coronavirus 2); hRV/SARS-Like (Rhinovirus/Coronaviruses); hRV/RSV (Rhinovirus/Syncytial); hRV/Influenza (Rhinovirus/Influenza); hRV/ADE (Rhinovirus/Adenovirus); hRV/hPVI (Rhinovirus/parainfluenza); hRV/MPV (Rhinovirus/Metapneumovirus).

Even though most of the clinical symptoms were related to a single infection (i.e. pneumonia, bronchiolitis, wheezing, sneezing, cough, runny nose/rhinorrhea, dyspnea/tachypnea, and O2 use), part of the respiratory symptoms may be related to higher prevalence of chronic asthma in patients with thoracic changes (i.e. acute reticular infiltrate, x-ray opacification, pulmonary hyper transparency, pleural effusion) and consequent intermittent mandatory ventilation (IMV) were more frequent in patients with co-infection (Figures 8 and 9(a,b)).

Different co-infections were analyzed; the results are shown on Figure 9. The (Figure 9(a,b)) required the use of NIV and IMV compared to the other co-infections. Furthermore, hRV/MPV and hRV/hBoV induced most of the clinical symptoms observed in the patients in addition to the need for interventions to improve their breathing (Figure 9(a,b)).

**Figure 10.**
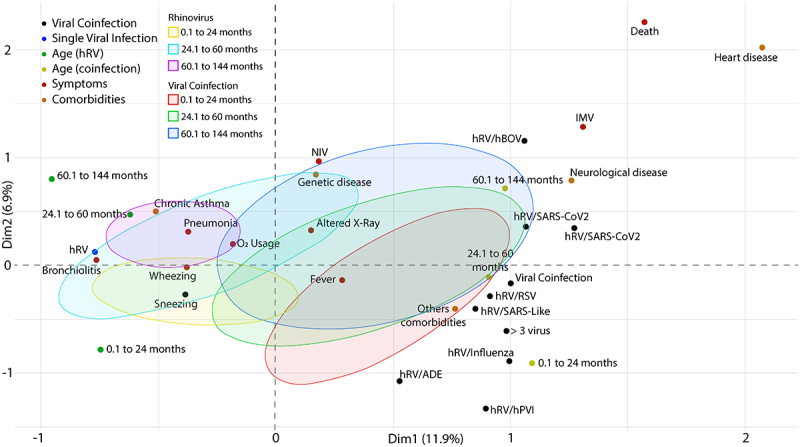
Multiple correspondence analysis (MCA) of patients with a single rhinovirus () infection and with co-infections with other respiratory viruses according to their age. Abbreviations: C/R (runny nose/rhinorrhea), D/T (dyspnea/tachypnea), NIV (non-invasive ventilation), IMV (intermittent mandatory ventilation). hRV/BoV (rhinovirus/Bocavirus); hRV/SARS-CoV-2 (rhinovirus/Coronavirus 2); hRV/SARS-Like (rhinovirus/Coronaviruses); hRV/VSR (rhinovirus/Syncytial); hRV/Influenza (rhinovirus/Influenza); hRV/ADE (rhinovirus/Adenovirus); hRV/hPVI (rhinovirus/parainfluenza); hRV/MPV (rhinovirus/Metapneumovirus).

Different ages of patients with single infection and co-infection (Figure 10). The MCA indicates a clear separation among the groups of patients with a single infection, their ages, and the different co-infections, with a representativeness of 18.8% of the variance between the two dimensions (Dim1 = 11.9%, Dim2 = 6.9%).

All co-infections induced pulmonary alterations, but a greater number of patients with hRV/MPV and hRV/SARS-Like (Figure 10(b)) required the use of non-invasive ventilation (NIV), while a greater number of patients with hRV/hBOV and hRV/PVI.

Age-related symptomatologic differences were more prevalent in patients with a single infection, while patients with co-infections had similar conditions regardless of age (Figure 10). The condition of patients with a single infection aged 24–60 months was related to a greater induction of pulmonary alterations, bronchiolitis, and most clinical symptoms (Figure 10), while patients aged 0.1 to 24 months had chronic asthma, wheezing, cough, runny nose/rhinorrhea and sneezing, while patients aged 60.1 to 144 months had pneumonia, dyspnea/tachypnea, and needed to use O2.

## Discussion

During the epidemiological surveillance for the detection of SARS-CoV-2 in children with severe acute respiratory infection. Despite this being a period of social mitigation to contain the spread of the COVID-19 pandemic, rhinovirus was the virus most detected, the authors detected this results in the present study.

Wu and collaborators [20] described that the Guangzhou government took mitigation measures to stop the COVID-19 pandemic and was a success because in addition to slowing COVID-19, it also contained common infectious diseases such as influenza and pneumonia, but for any reason was not contained the rhinovirus infections.

For Teo and collaborators [21], seasonal viruses were suppressed during the COVID-19 pandemic due to preventive measures such as mask wearing, social distancing, lockdown, but did not contain the rhinoviruses that persisted throughout the pandemic, as they are known because they are effectively transmitted by the airways and by fomites because they survive longer on surfaces and resist alcohol-based disinfectants [22], and therefore continued to infect children and adults throughout the pandemic period.

All hospitalized children followed the protocol for SARI and the clinical symptoms most detected in patients at the time of admission were cough, dyspnea, rhinorrhea, and wheezing, among others. Known as the common cold, often presents with the clinical syndrome of rhinorrhea, nasal congestion, sore throat, cough, headache, and malaise [18]. SARI due to rhinovirus, was associated in greater proportion with bronchiolitis, pneumonia and asthma attacks, acute respiratory failure (ARI), it is suggesting that rhinovirus may be associated with severe respiratory disease in children of the studied age group.

A study from Tunisia analyzed samples of 271 children with aged between 1 day and 144 months. The researchers detected that the least one respiratory virus in 170 positive cases (62.7% of SARI cases) and 144 samples positive for HEV coinfection, representing 61.1% of viral coinfection cases and 57 cases (33.5%) were positive [23].

A study from Mexico [24] described the clinical and molecular features of infection in the Mexican population; and explored the association between type and illness. A total of 5662 participants were analyzed and 1473 (26%) tested positive. A total of 485 participants (32.9%) were children and the total them, 35.5% of children were outpatients and 23.5%, respectively, were hospitalized (non-ICU); 15.3% of children were in the emergency room; and 18.8% of children were in the ICU. One hundred thirty-seven (28.2%) children had a coinfection. In children, 79% (383) of cases occurred in those ≤5 y of age. The most common comorbidities in children were congenital syndromes, occurring in 89 (18.4%) children.

Hanchi et al.. (2022) [25], studied 902 respiratory specimens from Marroccos. Human rhinovirus was detected throughout the year, and more prevalent in winter during COVID-19 (*p* = 0.0002). The occurrence of SARI was higher in male children (*n* = 525, 58.2%). More than half of the patients were less than 6 months old (*n* = 485, 54.2%), with no significant difference between the two periods before and during COVID-19. The main common reason for hospitalization was severe bronchiolitis with respiratory distress, mostly in children under one year old (*p* < 0.05).

Leotte and collaborators (2016) [26], diagnosed 755 patients from Brazil with SARI in 2012 and 2013. The most frequently detected viruses were 37%; there were 162 cases positive for over the study period: 83 (51%) in 2012 and 79 (49%) in 2013, with 282 cases of other respiratory viruses’ infections during the same period. A comparison of the clinical characteristics of mono-infected patients with co-infected patients was performed, and no relation between viral co-detection and disease severity (*p* = 0.717) was found. As co-infection with alone was not related with severe disease, a second analysis was performed comparing disease severity between co-infected patients and patients infected with other CRVs. Neither the presence nor absence of co-detection was associated with disease severity (*p* = 0.196).

In the present study, all patients included were followed up until the outcome of the clinical picture of SARI hospitalized in the PICU and the chronic asthma was comorbitive more frequently with (30.71%) for patients with a single infection (*p* < 0.05). The percentage of SARI found in this study was clearly higher than that found by the other researchers described; this higher number can be justified by the origin of the collected samples, considering that all samples in this study come from patients in ICU. On the other hand, these changes could be explained by the impact of the implementation of preventive measures related to the COVID-19 pandemic on the transmission of respiratory pathogens in children.

The seasonality is a variable that greatly influences the viral clinical manifestation. A study developed in Finland observed that the incidence of rhinovirus in children with age between 0 and 14 y old, remained stable between January 2019 and March 2020, observing a decrease during the lockdown (March 2020). After lifting the lockdown (June 2020), rhinovirus rates returned to normal levels and remained stable. The lockdown temporarily stopped the circulation of almost all respiratory pathogens in the spring of 2020. However, researchers have identified that the rhinovirus has a high potential for contamination in children [27].

When assessing seasonality, scholars state that the rhinovirus circulates throughout the year in climatic regions with occasional peaks in autumn and winter [28]. A study carried out in Croatia diagnosed that during autumn and spring, the rhinovirus manifested more frequently. Severe rhinovirus infection is more common in winter due to viral inefficiency of immune factors at low temperatures [29].

The percentage of children with rhinovirus according to age group did not differ much between the years 2018 to 2021. Data show that in 2020 a percentage of children between 30% and 50% were positive for the rhinovirus. According to the clinical manifestation, researchers estimate that the rhinovirus can cause acute wheezing and asthma exacerbation in children, especially during the winter [30].

Studies demonstrate that the clinical manifestation of the rhinovirus remained unchanged during the pandemic. However, rhinovirus levels were endemic in August 2020, mainly due to the absence of influenza and respiratory syncytial virus, presenting a peak of positivity with values close to pandemic levels. This peak can be explained by the variety of the mode of transmission compared to SARS-COV-2, high survivability on surfaces or the lack of an envelope that could provide protection against disinfectants and soap [31].

In the present study, the increased levels of clinical manifestation of rhinovirus corroborate data from other authors cited in this discussion. We observed that the seasonality of co-infection cases, the number of cases was similar between 2020 (49.53%) and 2021 (51.47%), with infection peaks in April (35.84%) of 2020, and in March (16.66%), May (14.81%) and August (15.09%) of 2021, winter period on Brazil.

After assessing seasonality, another variable that interferes with the viral clinical manifestation is the presence of comorbidities. In the present study, bronchiolitis was the comorbidity that most affected the children who participated in this study. Researchers consider rhinovirus to be the most common pathogen associated with bronchiolitis, contributing to the development of long-term sequelae such as wheezing and asthma in the pediatric population [32].

Studies indicate that 85% of children hospitalized with rhinovirus had bronchiolitis and 50% of hospitalized children had fever. When evaluating the hospitalization records of these children, it was possible to observe that, on average, the children were hospitalized for 99 d, with 21% requiring non-invasive mechanical ventilation and 92.86% requiring oxygen therapy [33].

It was observed that during the COVID-19 pandemic, hospitalizations for bronchiolitis did not occur significantly. Furthermore, between 2020 and 2021, children were essentially infected by rhinovirus and this viral type was responsible for 21% of patients with bronchiolitis [34]. According to Peris [35], the rhinovirus was the etiological agent that most caused bronchiolitis between 2020 and 2021. Curatola and collaborators [36] reported that during the pandemic, 39.4% of pediatric patients were admitted to the emergency service, presenting clinical manifestations of bronchiolitis.

Studies indicate that bronchiolitis is the most common acute viral infection of the lower airways in infants and the main cause of hospitalization and death from viral infections in Western countries. The number of cases of rhinovirus bronchiolitis between 2020 and 2021 was 87% and the use of ventilatory support was 37% [37].

In this study, bronchiolitis had a statistically significant value *p* < 0.001, where 57.51% of children had this clinical condition upon admission to the ICU, followed by pneumonia (29.41%).

The main cause of hospitalization in children is acute bronchiolitis and the most common pathogen of this clinical symptom is the rhinovirus, which is responsible for 14% of admissions for lower respiratory tract infection in pediatric intensive care units (ICU), classified as one of the most recurrent viral infections each year due to its genetic diversity with more than 150 types [38–41]. Paula et al. [40], studied bronchiolitis and found the most frequent clinical diagnosis in lower respiratory tract infections, and the pneumonia was the second most frequently found. Bronchiolitis, in most children, has an uneventful course and usually about 3% of cases require hospitalization in a ward and approximately 2–6% of hospitalized cases require admission to a PICU [32,41].

Another data found by us, which could justify the severity of the cases of children with, is the presence of comorbidities such as chronic asthma with a value of *p* < 0.05. Rhinovirus infection is usually mild and self-limiting but can cause bronchiolitis in infants, pneumonia in immunocompromised patients, and exacerbation of asthma or chronic obstructive pulmonary disease in patients, occurring in up to two-thirds of asthma exacerbations in children; the main risk factors for children with are clinical conditions of allergy and asthma [42].

In this study, viral co-infection with two or more than three viruses was also detected, representing a rate of 40.93% of the analyzed cases. The identification of infant bronchiolitis by obtaining peripheral blood samples and performing molecular marker analyses is of great utility for to reflect the host’s immunological response, allowing a better understanding of the pathogenesis of viral infection.

Vieira and collaborators [43] had analyzed the leukogram profile in patients with viral infection and the results showed significantly (Mann–Whitney test, *p* = 0.03) more leukocytes in infants with infection and respiratory viruses. Choi et al. [44] also found leukocytosis in infants with infection compared with other respiratory viruses (HRSV, influenza virus, parainfluenza virus, human coronavirus, human bocavirus, human metapneumovirus). In this study, children with aged between 0 and 24 months had higher levels of lymphocytes (*p* < 0. 05).

Authors have studied that there is an increase in D-dimer levels in co-infection with adenovirus [45–48]. Similar results were found in the D-dimer levels of this work, in children aged 0–60 months with viral infection. This study also analyzed lower platelet levels in patients aged 60.1 to 144 months. Publications [49,50] found a deceased platelet count associated with virus coinfection.

SARS‐CoV‐2 infection is also associated with lymphopenia, prolonged prothrombin time (PT), high levels of LDH, ALT, AST, D‐dimer, C‐reactive protein (CRP), and troponin, along with neutrophilia and eosinophilia [51]. On the other hand, the coinfection with two viruses can either alleviate or increase the severity of the disease. A common consequence in such cases is viral interference. During viral interference, one virus competes with the other, interfering with the replication mechanism [52].

Louie and collaborators [38] also described that the rhinovirus may be associated with severe respiratory disease in children admitted to the ICU, since 80% of the cases studied by them, demonstrated on radiograph alterations in the lower respiratory tract, more than half of the children required mechanical ventilation and the mean length of stay was 7 d.

The ability to identify virus infection in patients with respiratory tract infection is potentially useful if validated, and more generally the investigation of these ratios in the discrimination of bacterial and viral would be valuable. Future work should focus on patient cohorts in need of new biomarkers and avoid comparisons between infected and healthy individuals. Overall, these biomarkers warrant further recognition and study in infectious disease.

## Conclusion

In these studies, it was revealed that despite social mitigation during the pandemic period, rhinovirus was the most frequent virus among children. The higher prevalence of rhinovirus compared to the lower prevalence of the other viral types addressed in this study can be explained by the fact that the rhinovirus is transmitted through the air and is associated with fomites, being able to survive for long periods on inanimate surfaces and present resistance to the action of alcohol. In this way, its viral particles remain viable and capable of infecting human organisms, even with health surveillance measures during the pandemic.

Another factor that favors the high frequency of viral circulation is the capacity for clinical manifestation during climatic variations, therefore not suffering great interference from seasonality. Some hematological parameters were evaluated, and leukocytosis was determined when a viral type was diagnosed in the children studied. In general, the clinical manifestation of the rhinovirus is mild, however, when in co-infection with other viral types or when associated with comorbidities, mainly bronchiolitis, pneumonia, acute respiratory failure and chronic asthma, the rhinovirus can be severe in the clinical manifestations of children in age group of the present study.

## Supplementary Material

Supplemental Material

## Data Availability

Data sharing is not applicable to this article as no new data were created or analyzed in this study.
