# Supplementary material for: Rhinovirus infection and co-infection in children with severe acute respiratory infection during the COVID-19 pandemic period
Source: Virulence. 2024 Feb 21;15(1):2310873. doi: 10.1080/21505594.2024.2310873 (PMC10885176; doi:10.1080/21505594.2024.2310873)
Supplement: Supplemental Material [file KVIR_A_2310873_SM7588.docx]

**
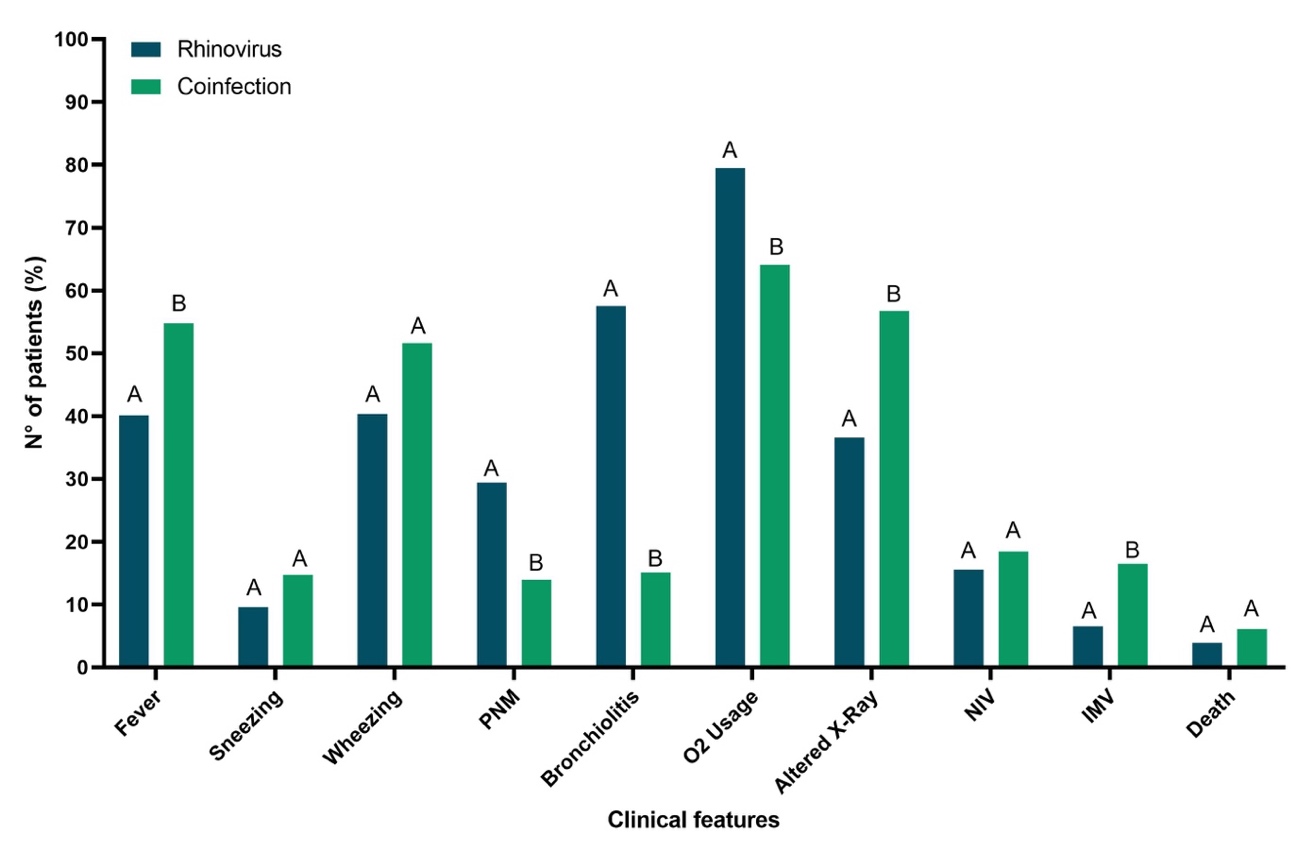
**

**Figure 1.** Clinical outcomes of children with single viral infection with Rhinovirus (hRV), and co-infection with hRV and another respiratory virus. Abbreviations: C/R (runny nose/rhinorrhea), D/T (dyspnea/tachypnea), PNM (pneumonia), AVB (acute viral bronchiolitis), NIV (non-invasive ventilation), IMV (intermittent mandatory ventilation). Capital letters indicate statistically significant differences (*p* < 0.05) in symptoms among patients with single infection and co-infection.


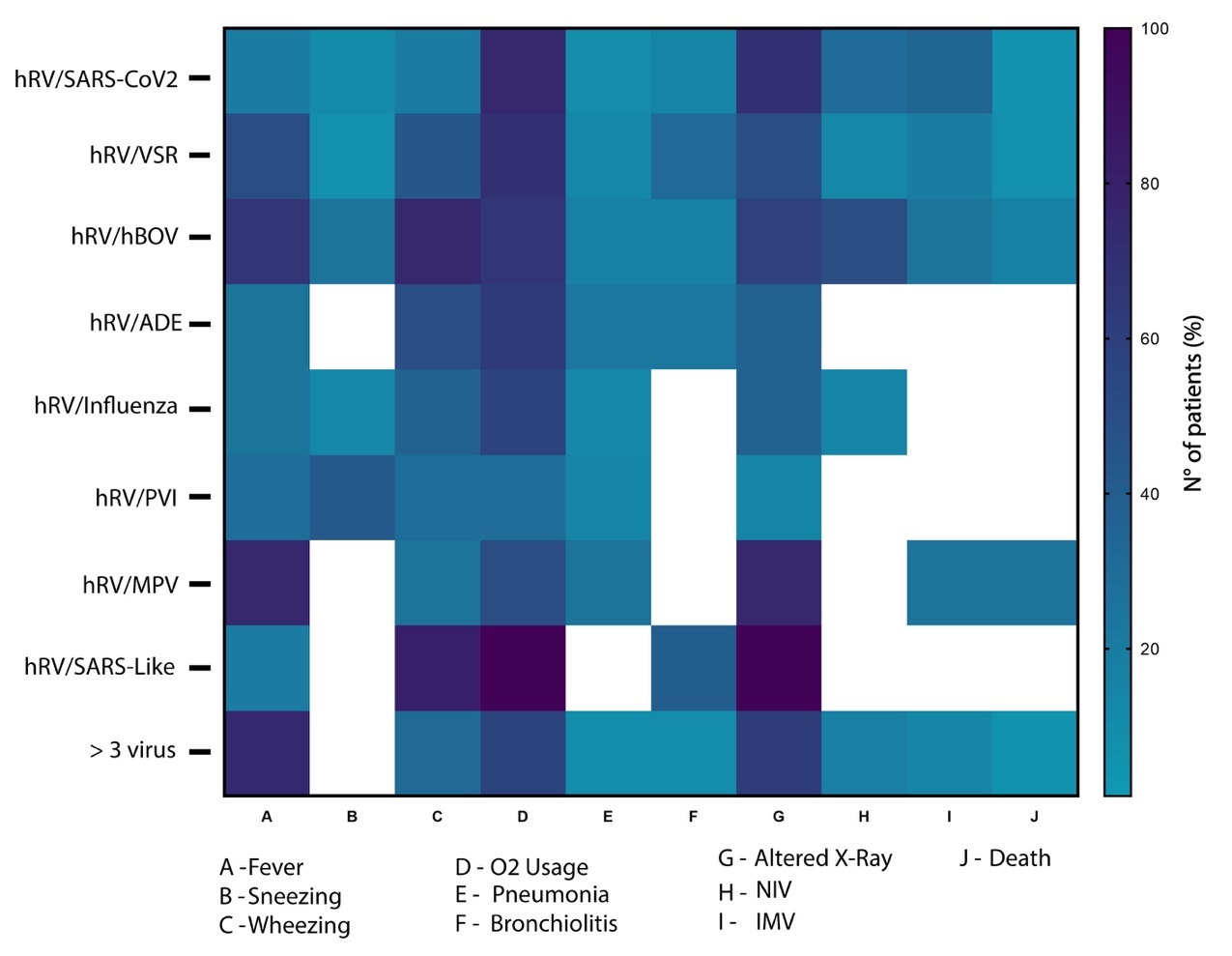


**Figure 2.** Clinical outcomes of children with different co-infections with hRV and another respiratory virus according to their age. Abbreviations: NIV (non-invasive ventilation), IMV (intermittent mandatory ventilation).
